# The predictive effect of extrinsic motivation on English online learning engagement

**DOI:** 10.3389/fpsyg.2025.1612002

**Published:** 2025-08-29

**Authors:** Zhixin Xu

**Affiliations:** College of Foreign Languages, Nanjing University of Aeronautics and Astronautics, Nanjing, China

**Keywords:** extrinsic motivation, online learning engagement, self-determination theory, hybrid teaching environment, college English

## Abstract

This study investigates the predictive role of extrinsic motivation on online learning engagement among Chinese non-English-major undergraduates, framed within Self-Determination Theory (SDT). Through a questionnaire survey of 472 students in hybrid teaching environments, the research examines the interplay between extrinsic motivation subtypes (external, introjected, identified, and integrated regulation) and multidimensional online engagement (behavioral, cognitive, emotional, and social). Results reveal that extrinsic motivation is predominantly manifested through autonomous forms (identified and integrated regulation), while overall online engagement remains moderate, with social engagement lagging significantly. Correlation and structural equation modeling analyses demonstrate that all extrinsic motivation subtypes positively predict engagement, with introjected regulation (less autonomous) exhibiting the strongest predictive power. These findings validate the continuity of motivational regulation in SDT and highlight the context-specificity of extrinsic motivation’s effects, providing theoretical insights for understanding motivation-engagement dynamics and pedagogical implications for optimizing hybrid teaching policies and motivational strategies in global foreign language contexts.

## 1 Introduction

The past decade has witnessed a profound transformation in teaching paradigms globally, marked by the deep integration of information technology into education. This shift has given rise to hybrid teaching, defined as a strategic integration of online self-directed learning with traditional offline instruction, which presents universal challenges for educators and learners (He, 2014; Chiu et al., 2021). Within this context, understanding the motivational drivers of online learning engagement has become a critical area of research, particularly in foreign language education where engagement is pivotal for success.

Online learning, as a highly context-dependent learning activity, necessitates greater learner motivation and self-regulated learning capabilities (Schunk et al., 2008; Zhang and Mou, 2013; Schwab and Somerville, 2022). However, existing literature reveals contradictory findings regarding motivational determinants of online engagement. While intrinsic motivation is widely acknowledged as a primary catalyst for sustained participation (e.g., Gao et al., 2015; Eom, 2019; Peng and Fu, 2021; Cheng, 2023; Xue, 2023), empirical evidence concerning extrinsic motivation remains highly contested. Some studies associate it with psychological resistance (e.g., Cho and Heron, 2015; Ye et al., 2022; Zeng et al., 2023) while others attribute increased participation to extrinsic rewards (e.g., Gao et al., 2015; Wu et al., 2020; He and Zhou, 2022; Zhou and Zhang, 2024). This inconsistency underscores an underdeveloped understanding of extrinsic motivation’s predictive power regarding online engagement. Second, existing literature largely overlooks potential stage-specific variations in online learning behaviors across different extrinsic motivation subtypes. These gaps limit both theoretical advancements and practical applications in hybrid teaching environments.

**Figure 1:**
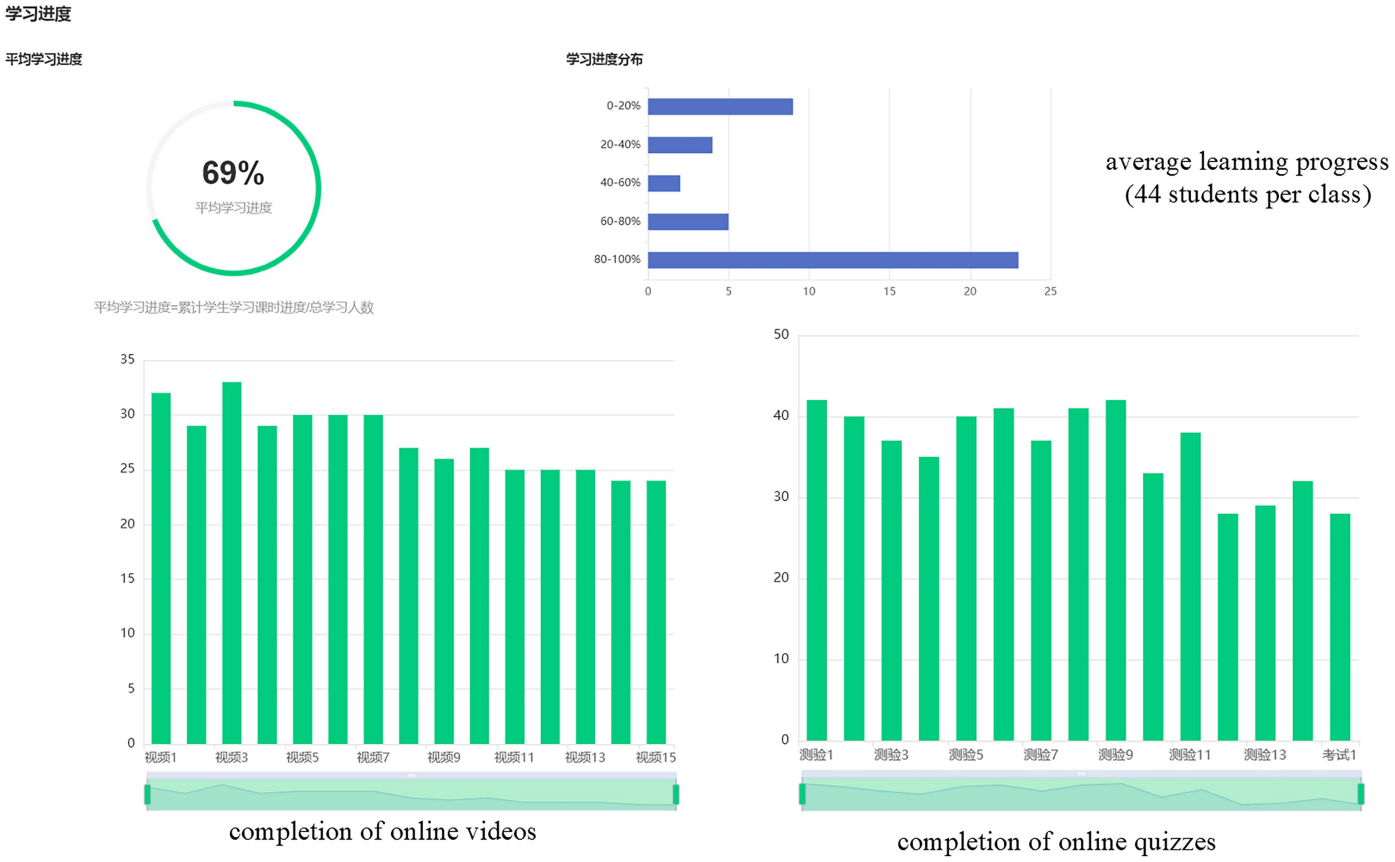
Online learning based on MOOC records.

China, the largest EFL (English as a Foreign Language) market worldwide, has recommended hybrid College English courses for all non-English majors under the 2020 National College English Teaching Guidelines (University Foreign Language Teaching Steering Committee of Ministry of Education, 2020). This policy context, coupled with the collectivist–high-stakes examination culture (Su, 2015), yields a unique motivational ecology where extrinsic drivers (e.g., grades, CET^1^ certificates, job market pressure) dominate. Field data collected in Autumn 2023 at the research site from Chinese hybrid EFL classrooms further reveal a paradoxical “participation without involvement” phenomenon: while most actively completed asynchronous tasks (e.g., video watching, quizzes), synchronous interactions (e.g., online discussions, group collaborations) remained superficial, with over 60% of students responding only to mandatory prompts (Figure 1). Consequently, understanding how extrinsic motivation functions within Chinese non-English-major undergraduates’ online English learning constitutes not only a contextual gap in global educational research but also a critical inquiry into how macro-level policies and cultural values shape micro-level motivation-engagement dynamics in hybrid EFL settings.

The above-mentioned issues, therefore, warrant further research on the predictive effects of extrinsic motivation subtypes on multidimensional online engagement in hybrid teaching contexts. This study therefore employs Self-Determination Theory (SDT) as its conceptual framework to examine how extrinsic motivation subtypes differentially predict online English-learning engagement among Chinese non-English-major undergraduates. By integrating theoretical rigor with contextual specificity, this investigation aims to generate evidence-based insights for optimizing hybrid teaching models and fostering effective online learning engagement in in global foreign language education.

## 2 Literature review

### 2.1 Self-determination theory

Self-Determination Theory (SDT) provides a granular lens for unpacking extrinsic motivation, distinguishing it from binary models by framing motivation as a continuum of autonomy (Ryan and Deci, 2000). This framework is particularly suited for hybrid learning contexts where motivation is fluid and context-dependent (Noels, 2002), and aligns with the “person-in-context” perspective emphasizing situational influences on L2 motivation (Dörnyei and Ushioda, 2011).

Self-determination Theory (SDT), initially proposed by Deci and Ryan (1985), posits that individuals’ reason for performing a given activity can be understood through the lens of their inherent psychological needs and their recognition of environmental factors. SDT therefore is a situational motivation theory based on the premise of individual autonomy and it conceptualizes motivation as a continuum ranging from amotivation through extrinsic motivation to intrinsic motivation, progressing from low to high autonomy (Ryan and Deci, 2000):

Amotivation: a state characterized by individuals’ perception of the irrelevance or futility of their actions.

Intrinsic motivation: regarded as a fundamental driver in educational contexts, emerges from learners’ natural curiosity and inherent interest in the learning process (Dörnyei, 2003).

Extrinsic motivation: defined as behavior aimed at achieving separable outcomes (Ryan and Deci, 2000), typically originates from such external incentives as academic rewards, financial compensation, or avoidance of negative consequences.

Noels (2002) further subdivides extrinsic motivation into four regulatory stages along an autonomy continuum: (1) external regulation, where behavior is externally determined to satisfy external demands like obtaining rewards or avoiding punishments; (2) introjected regulation, characterized by internal pressures to maintain self-esteem or avoid guilt; (3) identified regulation, marked by more self-determined recognition of the importance and the value of learning behavior; and (4) integrated regulation, where external motivations are fully assimilated into one’s self-concept, resembles intrinsic motivation in its self-governance but differs fundamentally since the activity is pursued not for inherent enjoyment but as an expression of self-identity (Noels, 2002).

This continuum reflects a progressive increase in autonomous regulation from external to integrated forms of extrinsic motivation (Ryan and Deci, 2000). SDT’s granularity offers a theoretical foundation for examining the complex interplay between learner motivation and learning behavior in hybrid teaching environments, particularly in understanding how different stages of extrinsic motivation influence online learning engagement. However, the present effort to explore L2 motivation from the SDT perspective is mainly at a theoretical level and inadequate in empirical studies (Li et al., 2022).

### 2.2 Online learning engagement

Online learning engagement, a critical indicator of hybrid teaching effectiveness, has evolved through three conceptual phases.

Initially, Tyler (1949) established the cost-reward paradigm based on workforce engagement studies, positing that increased personal investment in professional activities yields proportional rewards. This conceptual framework was subsequently adapted to educational contexts by Schaufeli et al. (2002), who reconceptualized engagement as a learning-specific state comprising three core components: vitality, dedication, and attention. Building on Bloom’s taxonomy of educational objectives (Bloom et al., 1956), Fredricks et al. (2004) expanded this model through longitudinal studies, establishing a four-dimensional framework that has become foundational in contemporary educational research. Their operationalization defines learning engagement as “the quality of active participation and sustained positive orientation that learners demonstrate in educational activities” (Fredricks et al., 2004, p. 62), encompassing behavioral, cognitive, emotional, and social dimensions–a classification subsequently validated through multiple replication studies (e.g., Fredricks et al., 2016; Philp and Duchesne, 2016).

Within hybrid learning environments, online learning engagement emerges as a distinct psychological construct characterized by four integrated dimensions:

(1) Online behavioral engagement: a high level of learner engagement in online learning activities including attention, effort and persistence, which can reflect learners’ involvement in the hybrid teaching environment (Fredricks et al., 2004).
(2) Online cognitive engagement: a high level of cognitive and psychological engagement of learners during online learning process, especially the cognitive indicators after deep thinking to understand and master online learning content (Ravindran et al., 2005).
(3) Online emotional engagement: emotional reactions that learners exhibit during the online learning process, encompassing emotional states ranging from academic curiosity to content-related frustration (Pekrun et al., 2011).
(4) Online social engagement: learner’s online interaction and cooperation with teachers or peers around the learning content (Li and Yu, 2015; He and Zhou, 2022).

Empirical studies have demonstrated that this multidimensional construct serves as a robust predictor of academic achievement in blended learning environments (Li and Yu, 2015), while also functioning as a key quality indicator for evaluating hybrid course design effectiveness.

### 2.3 Motivation and online learning engagement

Online learning engagement, as a dynamic construct akin to learner motivation, is profoundly shaped by contextual determinants. Empirical evidence suggests that this multidimensional phenomenon may be influenced by such factors as learner characteristics, teachers, peers, and online learning platforms (e.g., Gao et al., 2016; Wan et al., 2021; He and Zhou, 2022). Motivation, which encompasses both the direction and magnitude of human behavior (Williams and Burden, 2000), extends beyond mere interest arousal - it sustains effort and goal-directed persistence. Intrinsic motivation drives learners’ autonomous choices in content, style, and time investment, whereas extrinsic motivation facilitates deliberate behavioral regulation (Wolters and Benzon, 2013), collectively contributing to learning outcomes.

The hybrid teaching mode, characterized by strong situational complexity and weak behavioral controllability, necessitates amplified motivational engagement. Unlike traditional language classrooms, hybrid teaching mode may capitalize on technological affordances to stimulate intrinsic motivation to enhance online learning engagement (Lepper and Malone, 1987; Martens and Kirschner, 2004). Extrinsic motivation, in turn, can further prompt students to invest greater effort and persistence in completing learning tasks (Reindl et al., 2020). While numerous studies have validated and supported the conclusion that extrinsic motivation can promote online learning engagement (Adnan, 2020; Laili and Nashir, 2021), consensus remains elusive regarding the nature of this relationship. A notable gap in existing research is the neglect of learner motivation as an emotional variable that fluctuates with individual cognition, emotion, and environmental interactions, therefore leaving unexplored potential differences in the relationship between online learning engagement and motivation across distinct stages.

The online learning behavior is facilitated by the joint effect of students’ intrinsic motivation and extrinsic motivation. In the post-pandemic era, as students grow more familiar with hybrid teaching, the intrinsic curiosity triggered by network technology itself may gradually weaken. Instead, frustrations or anxieties caused by such factors as technical problems, network failures, and learning loneliness during the online learning process will seriously affect students’ extrinsic motivation for online learning (Artino, 2008).

This motivational paradox intensifies in China’s college EFL context due to cultural-linguistic disparities, authentic communication deficits, and the restrictive multiple-choice assessment paradigms (Su, 2015; Seker, 2016). Compounding these challenges, non-English-major undergraduates in this context typically exhibit stronger extrinsic motivation relative to intrinsic motivation (Liu, 2010; Yu et al., 2018).

An in-depth exploration is therefore of necessity on the relationship between students’ online learning engagement and their extrinsic motivation at different stages. Such examination promises dual academic contributions, that is, enriching theoretical understanding of motivational scaffolding in hybrid teaching environment, while informing practical improvements in curricular resource management and digital task design for EFL pedagogy.

## 3 Research methods

### 3.1 Research question

Existing studies have presented conflicting evidence regarding the relationship between extrinsic motivation and online learning engagement in hybrid teaching environment. While some scholars posit a positive correlation (e.g., Zeng et al., 2023), others report insignificant or context-dependent associations (e.g., He and Zhou, 2022). Particularly noteworthy is the lack of in-depth exploration on how this relationship evolves across different stages of extrinsic motivation. To address these theoretical gaps, this quasi-experimental study investigates the dynamic interplay between extrinsic motivation and learning engagement among Chinese non-English-major undergraduates in a hybrid teaching environment.

The research questions are as follows:

(1) What constitutes the primary extrinsic motivators for non-English-major undergraduates in a hybrid EFL teaching environment?
(2) To what extent do these learners demonstrate behavioral, cognitive, emotional and social engagement in online English learning?
(3) How does different stages of extrinsic motivation predict online learning engagement?

And based on SDT and prior studies, the following hypotheses were derived:

*H1:* Extrinsic motivation will be predominantly manifested through more autonomous regulatory styles (identified and integrated regulation) rather than less autonomous ones (external and introjected regulation).

*H2:* Online learning engagement will be moderate overall, with social engagement scoring significantly lower than behavioral, cognitive, and emotional engagement.

*H3:* Each extrinsic-regulation subtype (external, introjected, identified, integrated) will positively predict overall online learning engagement.

*H4:* The predictive power of extrinsic motivation subtypes will differ, with more autonomous regulations (identified, integrated) exhibiting stronger predictive effects than less autonomous ones (external, introjected).

### 3.2 Participants

Participants were purposively recruited from a “Project 211^2^” and “Double First-Class^3^” university, which represents China’s top-tier higher education institutions (see footnotes 1–2). This sampling ensures the sample reflects the population most affected by national hybrid teaching policies.

The sampling proceeded in four stages.

Stage 1-population definition: All first-year non-English-major undergraduates enrolled in the compulsory College English IV course (hybrid mode for 2 semesters) across 15 colleges of the university (*N* = 3, 841).

Stage 2-stratification: the population was stratified by college (STEM vs. Social Science-Humanities) and gender to mirror university-wide proportions (61% STEM, 39% SSH; 56% male, 44% female).

Stage 3-recruitment: Using Wenjuanxing online survey platform, an invitation was sent (during mid-spring 2024) to 1,200 randomly selected students (proportionate to strata size) and obtained 512 informed-consent clicks.

Stage 4-screening and final sample: After excluding incomplete responses (*n* = 32) and careless responders (identical string >5 items, *n* = 15), the analytical sample comprised 472 students, representing 12.3% of the population with <2% deviation from the target gender/major ratio.

The final sample comprised 208 female (44.1%) and 264 male (55.9%) students aged 18–19 years (*M* = 18.7, *SD* = 0.43), representing diverse academic disciplines: 212 students (44.9%) from social sciences and 260 students (55.1%) from STEM fields. This gender and disciplinary distribution align with typical enrollment patterns in Chinese universities.

### 3.3 Research instruments

The questionnaire employed in this study includes three sections: personal information, English Learning Extrinsic Motivation Scale (ELEMS), and English Online Learning Engagement Scale (EOLES). Both scales use a Likert 5-point format (1 = strongly disagree to 5 = strongly agree) and all reverse questions are scored in reverse. A pilot study was conducted on 71 non-English-major undergraduates in advance.

The ELEMS was adapted from Black and Deci’s (2000) Adult Version of the Learning Self Regulation Questionnaire, grounded in Self Determination Theory. This instrument examines four dimensions of extrinsic motivation: external regulation, introjected regulation, identified regulation, and integrated regulation. The 16-item scale contains four items per dimension. Pilot testing (*N* = 71) demonstrated acceptable internal reliability: Cronbach’s *α* coefficients ranged from 0.72 to 0.81 across subscales, while confirmatory factor analysis indicated adequate model fit (*χ^2^* = 100.43, *χ^2^*/*df* = 2.35, *CFI* = 0.89, *TLI* = 0.91, *RMSEA* = 0.064, *SRMR* = 0.057).

The EOLES was modified from Deng et al.’s (2020) MOOC Engagement Scale (MES), measuring four engagement dimensions: behavior, cognition, emotion, and social interaction. The 12-item scale contains three items per dimension. Pilot data (*N* = 71) revealed satisfactory reliability (*α* = 0.71–0.85) and acceptable model fit indices (*χ^2^* = 39.21, *χ^2^*/*df* = 2.35, *CFI* = 0.93, *TLI* = 0.91, *RMSEA* = 0.057, *SRMR* = 0.035).

Notably, previous studies have identified significant associations between online learning engagement and demographic covariates including gender, discipline, and educational background (He and Zhou, 2022; Sun and Sun, 2011; Zhao and Yang, 2015). These variables were therefore incorporated as control variables in this study.

### 3.4 Data analysis

To address the three research questions, a two-phase analytical approach was implemented. First, descriptive statistics and correlation tests were analyzed using SPSS Statistics 28.0 to establish baseline characteristics of the variables. Subsequently, structural equation modeling (SEM) was performed with AMOS 26.0 to investigate the hypothesized predictive relationships between extrinsic motivation and online learning engagement, with maximum likelihood estimation employed for parameter calculation.

## 4 Research results

### 4.1 Common method biases

To address potential common method bias associated with self-reported measures, Harman’s single-factor test was implemented through exploratory factor analysis (EFA). The unrotated factor solution revealed five distinct factors with eigenvalues exceeding 1.0, collectively accounting for 61.03% of the total variance. Crucially, the first factor explained 35.47% of the variance, below the 40% threshold recommended by Podsakoff et al. (2003) as indicative of substantial common method variance.

Subsequent confirmatory factor analysis (CFA) of the measurement model demonstrated acceptable fit indices: *χ^2^* (*df* = 555) = 1487.42, *χ^2^*/*df* = 2.68, *CFI* = 0.91, *TL* = 0.93, *RMSEA* = 0.05. These psychometric properties, coupled with Harman’s test findings, collectively suggest that common method variance did not substantially threaten the validity of our measurement model.

### 4.2 Extrinsic motivation for participants’ English learning

The quantitative analysis revealed distinct patterns in extrinsic motivation dimensions among Chinese non-English-major undergraduates engaged in English online learning. Descriptive statistics demonstrated moderately high overall extrinsic motivation (*M* range = 3.49–4.07, *SD* range = 0.42–0.61), with the mean scores progressively increasing across the self-determination continuum: external regulation (*M* = 3.56), introjected regulation (*M* = 3.70), identified regulation (*M* = 3.91), and integrated regulation (*M* = 4.02).

To examine differences among the four extrinsic motivation subtypes, a one-way repeated-measures ANOVA was conducted, followed by Tukey’s *post hoc* tests (*N* = 472). Results showed a significant main effect of regulatory style [*F*(3, 1,413) = 87.62, *p* < 0.001, *η^2^* = 0.15]. *Post hoc* tests indicated:

Post hoc tests revealed that integrated regulation (*M* = 4.02, *SD* = 0.42) was significantly higher than identified regulation (*M* = 3.91, *SD* = 0.49) (*p* <.05); identified regulation was significantly higher than introjected regulation (*M* = 3.70, *SD* = 0.53) (*p* <.001); and introjected regulation was significantly higher than external regulation (*M* = 3.56, *SD* = 0.61) (*p* <.001). These results demonstrate a progressive increase in autonomy along the SDT continuum, which is consistent with Hypothesis 1.

This study further employed a two-way independent samples ANOVA with major and gender as grouping variables to examine subgroup differences in extrinsic motivation dimensions among participants. Statistical analysis revealed no significant between-major differences in extrinsic motivations (*p* > 0.05). However, gender-based comparisons demonstrated notable variations: female students exhibited significantly higher utilization of identified regulation [*F*(1, 470) = 6.02, *p* = 0.014, *η^2^* = 0.013] and integrated regulation [*F*(1, 470) = 4.51, *p* = 0.034, *η^2^* = 0.009] compared to their male counterparts. No significant gender differences emerged for external regulation [*F*(1, 470) = 1.12, *p* = 0.291] or introjected regulation [*F*(1, 470) = 2.34, *p* = 0.127], both representing less autonomous motivational forms.

### 4.3 Engagement in English online learning

The results showed that Chinese non-English-major undergraduates’ online English learning engagement exhibited a moderate overall level (*M* = 3.60, *SD* = 0.48), with dimensional mean scores distributed as follows: behavioral (*M* = 3.81, *SD* = 0.52), cognitive (*M* = 3.75, *SD* = 0.49), emotional (*M* = 3.64, *SD* = 0.57), and social interaction dimensions (*M* = 3.23, *SD* = 0.61). All scores fell between the scale anchors of 3 (“uncertain”) and 4 (“basically agree”), suggesting transitional engagement characteristics. The results therefore supported the H2.

A one-way ANOVA with Tukey’s *post hoc* analysis demonstrated non-significant differences among the emotional, cognitive, and behavioral dimensions (*p* > 0.05). However, the social interaction dimension showed statistically significant inferiority to other dimensions (*p* < 0.001, *η^2^* = 0.12), indicating substantially limited peer-to-peer and teacher-student communication patterns in virtual learning environments.

This study also examined group differences in online English learning engagement through a two-factor between-subjects ANOVA, with major (liberal arts vs. science/engineering) and gender as independent variables. The analysis revealed statistically significant main effects for both factors. Specifically, liberal arts students demonstrated substantially higher engagement levels (*M* = 4.32, *SD* = 0.71) compared to their science/engineering counterparts (*M* = 3.89, *SD* = 0.63), *F*(1, 470) = 5.37, *p* = 0.021, *η^2^* = 0.011. Similarly, female students exhibited significantly greater engagement (*M* = 4.25, *SD* = 0.69) than male students (*M* = 3.95, *SD* = 0.67), *F*(1, 470) = 4.05, *p* = 0.045, *η^2^* = 0.009. These findings suggest potential major and gender-based variations in online learning behaviors that warrant further investigation.

### 4.4 Correlation between extrinsic motivation and online learning engagement

The bivariate Pearson correlation analysis showed statistically significant positive associations between the four subtypes of extrinsic motivation (external regulation, introjected regulation, identified regulation and integrated regulation) and online learning engagement (Table 1). Notably, introjected regulation with lower autonomy demonstrated stronger correlations than other extrinsic motivation subtypes. These results provide preliminary evidence that extrinsic motivation may contribute positively to learning engagement in digital environments, contradicting the conventional dichotomy of extrinsic motivation as inherently detrimental (Murayama et al., 2010; Gao et al., 2016).

**Table 1:** Correlation of extrinsic motivation and online learning engagement (*N* = 472).

Variable	*M*	SD	A	B	C	D	E
A. external regulation	3.56	0.58	1				
B. introjected regulation	3.70	0.61	0.411**	1			
C. identified regulation	3.91	0.47	0.368**	0.415**	1		
D. integrated regulation	4.02	0.42	0.309**	0.368**	0.472**	1	
E. online learning engagement	3.60	0.48	0.278**	0.408**	0.323**	0.331**	1

**p < 0.01.

### 4.5 Predictive effect of extrinsic motivation on online engagement

**Figure 2:**
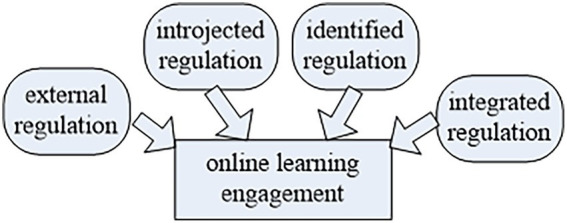
Explanatory framework.

This study utilized AMOS 26 to construct a structural equation model (SEM) examining the predictive relationships between four subtypes of extrinsic motivation (external, introjected, identified, and integrated regulation) and online learning engagement. Before construction, an explanatory framework (Figure 2) was established to explore the predictive relationships between the four subtypes of extrinsic motivation (external, introjected, identified, and integrated regulation) and engagement in hybrid teaching contexts.

**Figure 3:**
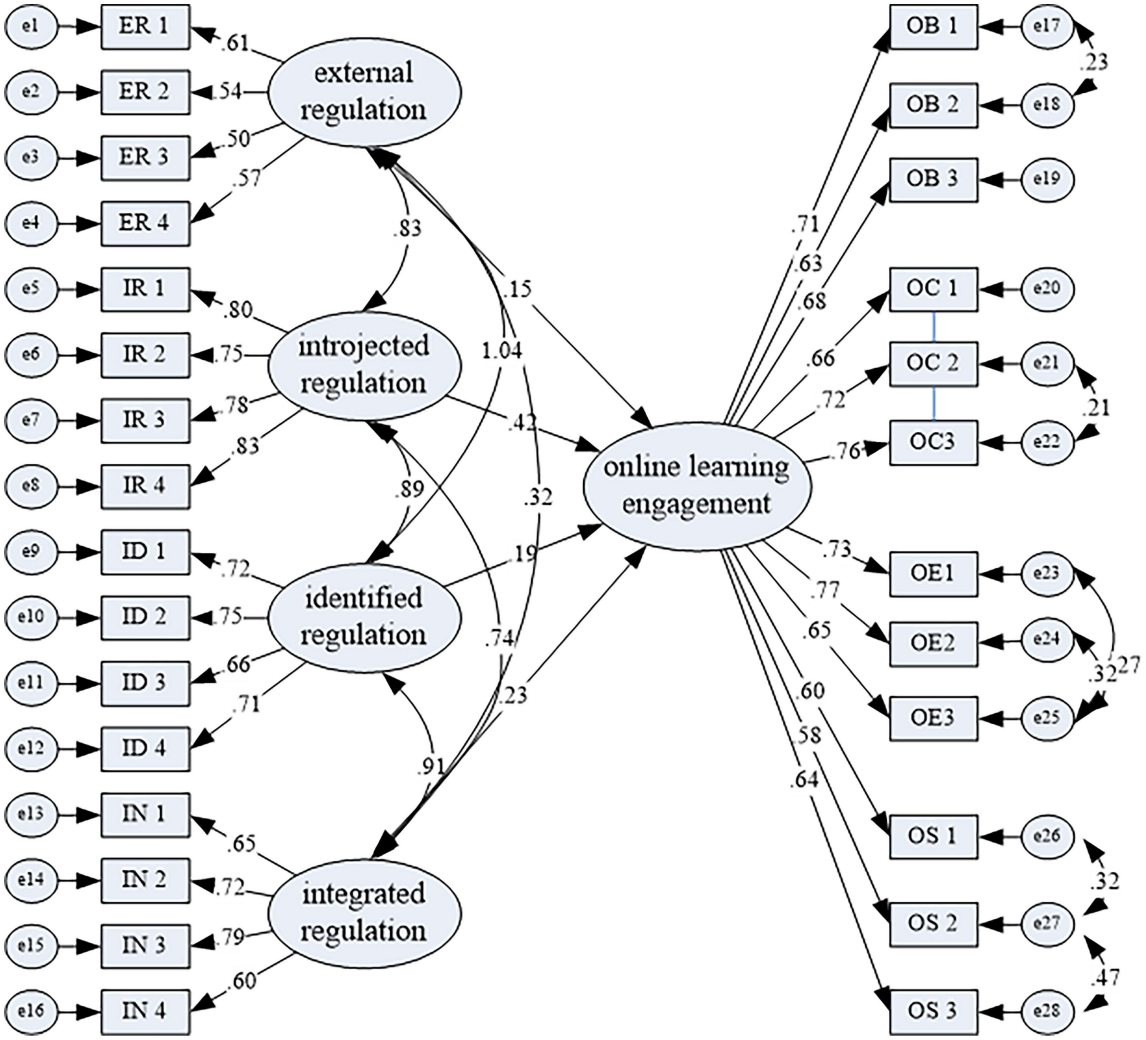
SEM of extrinsic motivation and online learning engagement.

The standardized path coefficients of SEM for extrinsic motivation subtypes predicting online Learning engagement were shown in Table 2. The measurement model (Figure 3) demonstrated acceptable to excellent fit based on multiple indices: *χ^2^* = 247.36 (*p* = 0.000), *χ^2^*/*df* = 2.52, *GFI* = 0.93, *AGFI* = 0.88, *CFI* = 0.95, *TLI* = 0.97, *RMSEA* = 0.040, *SRMR* = 0.05.

**Table 2:** Standardized path coefficients and fit indices of the SEM (*N* = 472).

Predictor	Path coefficient	*t*-value	*p*-value	95% CI
External regulation	0.15	3.21	<0.01	[0.07, 0.23]
Introjected regulation	0.42	7.89	<0.001	[0.35, 0.49]
Identified regulation	0.19	4.05	<0.001	[0.11, 0.27]
Integrated regulation	0.23	5.12	<0.001	[0.15, 0.31]

The results indicate that all four subtypes of extrinsic motivation have direct impacts on learners’ online learning engagement in a hybrid teaching context. In descending order of magnitude, introjected regulation exerted the strongest direct effect on engagement (*β* = 0.42, *p* < 0.001), followed by integrated regulation (*β* = 0.23, *p* < 0.001), identified regulation (*β* = 0.19, *p* < 0.001) and external regulation (*β* = 0.15, *p* < 0.01). The model explained 38% of the variance in online engagement, indicating a robust predictive utility.

Thus, the SEM results (Table 2) demonstrated that all extrinsic motivation subtypes positively predicted online engagement (supporting H3), but their predictive strengths contradicted H4: introjected regulation (less autonomous) exhibited the strongest effect (*β* = 0.42), followed by integrated (*β* = 0.23), identified (*β* = 0.19), and external regulation (*β* = 0.15). The above data lead to the following discussions.

## 5 Discussion

### 5.1 The overall situation of extrinsic motivation for online English learning

Guided by self-determination theory, this study categorizes extrinsic motivation into four types along the autonomy continuum to examine their influence on online learning engagement. Analysis reveals that non-English-major undergraduates demonstrated generally high extrinsic motivation scores across all four subtypes (*M* ≈ 4.00), with no significant differences across academic majors. This indicates that online English learning motivation in this population primarily operates through relatively internalized regulatory mechanisms, meaning students tend to engage not merely due to external pressures or self-imposed obligations, but also through personally valued goals and identity-congruent behaviors.

These findings align with prior studies (e.g., Gao et al., 2016; He and Zhou, 2022) demonstrating that online learners tend to be susceptible to multiple extrinsic motivators while developing heterogeneous regulatory approaches. The results further substantiate Ryan and Deci’s (2000) proposition regarding the situational nature of motivation, reinforcing the context-dependent characteristics of learner motivation. This phenomenon may stem from learners’ adaptive responses to environmental demands through strategic application of self-determination mechanisms.

Notably, identified and integrated regulations exhibited autonomy levels comparable to intrinsic motivation, challenging the conventional extrinsic-intrinsic dichotomy (Gao et al., 2015). The dynamic interplay between motivational forces and environmental factors creates an evolving motivational landscape where extrinsic motivations demonstrate fluid transformational potential (Hidi and Harackiewicz, 2000).

### 5.2 The overall situation of online learning engagement

The present study reveals that Chinese college students’ online English learning engagement demonstrates a moderate overall level, with observable variations across gender and academic disciplines. This finding diverges from Gao’s (2016) earlier finding of significantly lower engagement, potentially due to post-pandemic adaptation to hybrid pedagogies, which has fostered more positive perceptions of digital learning and enhanced willingness to engage in online academic behaviors.

Dimensional analysis discloses significantly weaker performance in emotional engagement and social engagement compared to cognitive and behavioral dimensions, aligning substantially with the dimensional patterns identified by He and Zhou (2022) but warrants deeper contextual exploration. First, in China’s high-stakes EFL context, where learning is often tied to standardized tests and grades (Su, 2015), students may prioritize individual task completion over social interactions, as the latter are not explicitly rewarded in assessment systems. This creates a “task-oriented” engagement pattern where social participation is perceived as peripheral to academic success. Second, the asynchronous nature of most hybrid classrooms in the sample reduces opportunities for spontaneous interpersonal exchanges and non-verbal communication cues, thereby constraining the development of socio-emotional connections (Wang, 2014). Third, technical barriers-such as unstable internet or poorly designed discussion forums-exacerbate feelings of isolation, as noted by Artino (2008), further suppressing social initiative.

Furthermore, the identified demographic variations (gender and major) corroborate existing study by Martin and Bolliger (2018) regarding individual difference factors. These differential engagement patterns collectively reinforce the conceptualization of online learning engagement as a context-dependent multidimensional construct shaped by the complex interplay of institutional ecosystems and learner characteristics.

### 5.3 The predictive effect of extrinsic motivation on online learning engagement

All four extrinsic motivation subtypes effectively and positively predict college students’ online English learning engagement, indicating that learners’ extrinsic learning motivation in hybrid teaching environments exerts a positive influence on their online learning engagement. These findings corroborate existing research asserting that extrinsic motivation facilitates enhanced online learning engagement (e.g., Hidi and Harackiewicz, 2000; Yang and Li, 2013; Gao et al., 2016; Ren, 2021; He and Zhou, 2022).

While extrinsic motivation remains crucial for stimulating learning engagement, the predictive strength does not strictly correspond to the degree of autonomous regulation, revealing the intricate nature of the effects from extrinsic motivation. The path coefficients demonstrate a descending order of predictive power: introjected, integrated, identified and external regulation. The dominance of introjected regulation (less autonomous) suggests that Chinese students are primarily driven by internal pressures like self-esteem maintenance or avoidance of guilt, even in temporally and spatially detached online environments. In practice, educators can leverage this by framing tasks as opportunities for personal growth rather than solely emphasizing external rewards. Additionally, providing timely, constructive feedback that frames effort as a marker of competence may reinforce healthy introjected motivation, reducing anxiety while sustaining engagement. Integrated regulation’s moderate impact highlights the value of aligning English learning with students’ long-term identities. Activities like reflective journaling or career-aligned projects could strengthen this connection.

Weak social engagement’s interplay with extrinsic motivation is particularly revealing. While extrinsic motivators like grades (external regulation) may spur task completion, they do little to foster meaningful peer interactions. To address this, educators could design collaborative tasks with shared rewards (e.g., group badges) or integrate social platforms that blend formal and informal communication.

In summary, these findings underscore that extrinsic motivation is not a monolithic construct but a continuum of regulatory styles, each interacting uniquely with engagement dimensions. Such evidence highlights the dynamic complexity of motivation influenced by external environmental factors. The primacy of introjected regulation in predicting engagement reflects the influence of China’s high-stakes educational culture, where self-esteem is tightly linked to academic performance (Su, 2015). Meanwhile, the weak social engagement highlights a critical opportunity: aligning social tasks with students’ motivational profiles - whether through structured accountability (introjected), value-based framing (identified), or initial rewards (external) - may bridge the gap between extrinsic motivation and holistic online engagement.

## 6 Conclusion

This study, grounded in Self-Determination Theory (SDT), investigates the current status of extrinsic motivation and online learning engagement among Chinese non-English-major undergraduates in hybrid teaching environments, while examining the correlation and potential predictive effects between extrinsic motivation and online learning engagement. Findings reveal that students’ extrinsic motivation in English learning is generally favorable, whereas their online learning engagement remains at a moderate level overall, with online social engagement requiring particular improvement. A significantly positive correlation exists between extrinsic motivation and online English learning engagement, with all four subtypes of extrinsic motivation demonstrating significant positive predictive effects–introjected regulation showing the strongest predictive power.

At the theoretical level, these findings demonstrate that extrinsic and intrinsic motivation do not exist in simple opposition, further validating Self-Determination Theory’s proposition that human behavioral motivations form a continuum. Additionally, the study confirms the contextualized SDT framework exhibits strong applicability and explanatory power in investigating learning motivation and online learning engagement within hybrid teaching environments in the context of Chinese college English education.

The pedagogical implications of this study are multifaceted. Given EFL learners’ characteristics, it is of necessity for College English teachers to systematically implement extrinsic motivation-driven strategies. First, priority should be given to the application of introjected regulation strategies, such as designing a “Learning Commitment Contract” system where students formulate and share personalized weekly learning goals, complemented by visual progress tracking tools. This approach leverages internal pressures while gradually encouraging self-determined behavior. Second, it may be useful to establish a diversified reward system through a “Credit Bank+” mechanism that converts online discussion quality and peer assessment participation into accumulable regular grades, providing immediate extrinsic incentives while scaffolding intrinsic value through collaborative learning. Third, the development of AI tutoring assistants capable of providing differentiated feedback based on students’ motivation types could personalize support and reduce pressure from real-time interactions, thereby nurturing sustained engagement.

To foster long-term engagement, extrinsic and intrinsic motivation should be strategically balanced. For instance, initial extrinsic rewards can be used to scaffold participation, while gradually integrating activities that emphasize autonomy, mastery, and personal relevance to cultivate intrinsic motivation. Additionally, creating opportunities for authentic communication and peer collaboration can address the deficit in social engagement while aligning with students’ integrated regulation, where English learning becomes part of their self-identity.

Several limitations should also be recognized. First, the sample was drawn from a single “Project 211” and “Double First-Class” university, which may limit the generalizability of findings to non-elite institutions or regions with different educational resources. Future research should include diverse samples across institutional types and geographic regions to enhance external validity. Second, the cross-sectional design and self-reported measures capture a snapshot of motivation and engagement, failing to account for their dynamic changes over time. Future research could employ longitudinal designs to track the dynamic interplay between extrinsic motivation and engagement over time, or mixed-methods approaches to capture richer qualitative insights into learners’ motivational and behavioral trajectories.

## Data Availability

The raw data supporting the conclusions of this article will be made available by the authors, without undue reservation.

## Footnotes

^1^College English Test, a national English proficiency test, hosted by the Department of Higher Education of the Ministry of Education of China, is mainly for non-English majors.

^2^The Project 211 refers to the construction project of about 100 key universities and key disciplines facing the 21st century. It was officially launched in November 1995 with the approval of the State Council.

^3^Double First-class University Project, also known as the “Double-First Class” initiative, mainly contains the university list published by Chinese authorities in 2018 for developing a number of world-class universities and disciplines by the end of 2050.
